# Supplementary material for: Content-rich biological network constructed by mining PubMed abstracts
Source: BMC Bioinformatics. 2004 Oct 8;5:147. doi: 10.1186/1471-2105-5-147 (PMC528731; doi:10.1186/1471-2105-5-147)
Supplement: Additional File 2 — The original results of the above study (non-essential files are deleted to keep the file size under the limit set by BMC bioinformatics). [file 1471-2105-5-147-S2.bz2 › chilibotAdditionalFile2/dip05/292ID9485448E1123/html/TRPA_TRPB.html]

 


 **TRPA** and **TRPB** 
  
Found 55 abstracts in PubMed, retrieved 05.  
 

 What does Google say? 
 PDF only 
| .edu only 

---

**Interactive relationship** (e.g. stimulation, inhibition, etc)

**Neutral relationship**- When a 4 bp overlapping leader ORF was placed in front of the successive  **trpB**  and  **trpA**  genes, the  **trpA**  gene was poorly expressed whereas the  **trpB**  gene was overexpressed.  Ref: 12097161 J Biochem Tokyo , 2002

**Non-interactive relationship** (e.g. studied together, co-existance, homology, etc.)

- The tryptophan synthases from Escherichia coli and Salmonella typhimurium are tetrameric enzymes, with an elongated  **TrpA** . **TrpB** . **TrpB** . **TrpA**  structure.  Ref: 11209753 Biol Chem, 2000
- Complementation analysis in Escherichia coli strains, with defined mutations in tryptophan biosynthesis, and in vitro enzyme activity data, with cloned  **TrpB**  and  **TrpA**  proteins, indicate these mutations result in a  **TrpA**  protein that is unable to utilize indole glycerol 3 phosphate as substrate.  Ref: 12011099 J Biol Chem, 2002
- The  **trpA**  and  **trpB**  genes encoding the tryptophan synthase subunits were cloned from the hyperthermophilic archaeon Pyrococcus furiosus.  Ref: 12435500 FEMS Microbiol Lett, 2002
- To explore the functional role of this region, we analyzed the effects of 19 single and double mutations in  **TrpB**  on the tryptophan synthase TSase and serine deaminase SDase activities of the TrpB2 dimer, either in the presence or in the absence of the  **TrpA**  subunit.  Ref: 11209753 Biol Chem, 2000
- Structural studies have identified residues 277 283 of  **TrpB**  as a potentially important region for the allosteric communication between the  **TrpA**  and  **TrpB**  subunits and for the transport of indole between their active sites through a hydrophobic tunnel.  Ref: 11209753 Biol Chem, 2000
- Gel filtration chromatography revealed that Tk  **TrpA**  was a monomer alpha, Tk  **TrpB**  was a dimer beta2, and Tk TS complex was a tetramer alpha2 beta2.  Ref: 11029579 Eur J Biochem, 2000
- Overexpression in Escherichia coli of the AT rich  **trpA**  and  **trpB**  genes from the hyperthermophilic archaeon Pyrococcus furiosus.  Ref: 12435500 FEMS Microbiol Lett, 2002
- The alpha and beta reaction activities of independently purified Tk  **TrpA**  and Tk  **TrpB**  were 10 fold lower than the respective activities detected from the Tk TS complex, indicating that during heat treatment, each subunit was necessary for the other to obtain a proper conformation for high enzyme activity.  Ref: 11029579 Eur J Biochem, 2000
- To overexpress broad kinds of GC rich genes in Escherichia coli, we examined how the structures of leader open reading frames leader ORFs affect the expression of GC rich genes, such as polA,  **trpA** , and  **trpB** , from Thermus thermophilus.  Ref: 12097161 J Biochem Tokyo , 2002
